# Genomic risk prediction of cardiovascular diseases among type 2 diabetes patients in the UK Biobank

**DOI:** 10.3389/fbinf.2023.1320748

**Published:** 2024-01-04

**Authors:** Yixuan Ye, Jiaqi Hu, Fuyuan Pang, Can Cui, Hongyu Zhao

**Affiliations:** ^1^ Program of Computational Biology and Bioinformatics, Yale University, New Haven, CT, United States; ^2^ Department of Chronic Disease Epidemiology, Yale School of Public Health, New Haven, CT, United States; ^3^ Department of Biostatistics, Yale School of Public Health, New Haven, CT, United States; ^4^ Department of Biostatistics, Shanghai Jiao Tong University, Shanghai, China; ^5^ Department of Immunobiology, Yale University School of Medicine, New Haven, CT, United States

**Keywords:** polygenic risk score, cardiovascular diseases, type 2 diabetes, risk prediction, age of onset

## Abstract

**Background:** Polygenic risk score (PRS) has proved useful in predicting the risk of cardiovascular diseases (CVD) based on the genotypes of an individual, but most analyses have focused on disease onset in the general population. The usefulness of PRS to predict CVD risk among type 2 diabetes (T2D) patients remains unclear.

**Methods:** We built a meta-PRS_CVD_ upon the candidate PRSs developed from state-of-the-art PRS methods for three CVD subtypes of significant importance: coronary artery disease (CAD), ischemic stroke (IS), and heart failure (HF). To evaluate the prediction performance of the meta-PRS_CVD_, we restricted our analysis to 21,092 white British T2D patients in the UK Biobank, among which 4,015 had CVD events.

**Results:** Results showed that the meta-PRS_CVD_ was significantly associated with CVD risk with a hazard ratio per standard deviation increase of 1.28 (95% CI: 1.23–1.33). The meta-PRS_CVD_ alone predicted the CVD incidence with an area under the receiver operating characteristic curve (AUC) of 0.57 (95% CI: 0.54–0.59). When restricted to the early-onset patients (onset age ≤ 55), the AUC was further increased to 0.61 (95% CI 0.56–0.67).

**Conclusion:** Our results highlight the potential role of genomic screening for secondary preventions of CVD among T2D patients, especially among early-onset patients.

## Introduction

Despite great advances in prevention and treatment in the past decades, cardiovascular diseases (CVD), the major cause of morbidity and mortality worldwide, remain a severe global health challenge (Vasan and Benjamin, 2016). CVD is even more prevalent among patients with type 2 diabetes (T2D) (International Diabetes Federation, 2015). According to a systematic review conducted in 2018, nearly 32.3% of T2D patients suffer from CVD worldwide, and it is the major cause of death among T2D patients, especially coronary artery disease (CAD) and stroke (Einarson et al., 2018). Besides, an increased risk for T2D is found to be followed by an increased risk for CVD (Fox et al., 2008).

As summarized previously (Wang et al., 2018), CAD, ischemic stroke (IS), and heart failure (HF) are the three main severe subtypes of CVD events. All three diseases have genetic components, with the heritability varying from 40% to 60% for CAD, 16% to 40% for IS, and 26% to 34% for HF (Bevan et al., 2012; Tayal et al., 2017; Lindgren et al., 2018; Aragam and Natarajan, 2020). More specifically, with the remarkable success of genome-wide association studies (GWASs) in recent years, hundreds of single nucleotide polymorphisms (SNPs) have been identified to be associated with CVD and its subtypes (Mehta, 2011; Carty et al., 2012; CARDIoGRAMplusC4D Consortium et al., 2013; Nikpay et al., 2015; Nelson et al., 2017; Malik et al., 2018; Shah et al., 2020). Polygenic risk scores (PRSs), the sum of risk alleles weighted by the effect sizes inferred from GWAS summary statistics, were subsequently constructed and have shown promise in predicting the onset of CVD as well as its subtypes (Khera et al., 2018). A few attempts have then been made to associate the PRS trained from CVD events occurrence among T2D patients, but with some limitations (Hong et al., 2020). These initial efforts were mainly hampered by three challenges: 1) the small sample size (less than 5,000) to train and test PRS; 2) less optimal risk prediction models for creating PRS; and 3) the same dataset used to construct PRS and test for prediction performance without validations.

With much larger studies and improved PRS methods available now, we set out to build a new PRS for CVD onset among T2D patients. Since several studies have shown that utilizing a meta-analytic strategy to build PRS can help better capture the genetic risk information (Inouye et al., 2018); also considering that CAD, IS, and HF are the major subtypes of CVD events with similar clinical implications and management (Hong et al., 2020; Ma et al., 2022) and there are strong genetic correlations among these three subtypes to potentially boost power of PRS (Dichgans et al., 2014; Verweij et al., 2017; Hong et al., 2020; Koyama et al., 2020), here we build a new PRS by combining the three “optimal” PRSs trained for each of these three CVD subtypes. With the newly-built meta-PRS_CVD_, we then comprehensively evaluate its prediction performance for CVD events among T2D patients. Besides, as several clinical variables have long been used in classic CVD risk predictions, we also evaluate the prediction performance of meta-PRS_CVD_ when integrated with those established clinical variables in predicting CVD occurrence. Furthermore, we investigate the roles of genetic and clinical risk factors in contributing to CVD among T2D patients with different onset ages, with the hypothesis that meta-PRS_CVD_ may have more predictive power among younger patients while CVD events among later-onset patients may be more driven by non-genetic factors.

## Materials and methods

### Study population

Our subjects are from the UK Biobank (UKBB), a large-scale prospective study established for investigating both genetic and non-genetic determinants of diseases among the middle-old aged population (Sudlow et al., 2015). Starting in 2006, 502,618 individuals aged 40–69 years were enrolled through 22 assessment centers throughout the United Kingdom (UK). Follow-up was conducted through linkages to Hospital Episode Statistics (HES), national death registries, and cancer registries. Specifically, HES used both the 9th and 10th revisions of the International Classification of Diseases (ICD9 and ICD10) to record diagnostic information, and OPCS-4 (Population, Census and Census Office: Classification of Interventions and Procedures, Version 4) to record surgical procedures. Currently available data from HES cover all hospital admissions from the NHS hospitals in England and Scotland from April 1994 to February 2021. And the death registries include all deaths in the UK up to January 2021.

We restricted our analysis to the white British participants where the ancestry was identified by a combination of self-reported ancestry and genetically confirmed ancestry based on principal component (PC) analysis of individuals’ genotypes (Bycroft et al., 2018). Additional exclusion criteria included discordance between reported and genotype inferred sex, poor heterozygosity or missingness, sex chromosome aneuploidy, and withdrawal of informed consent.

### Ascertainment of disease onset

Disease occurrences were identified by episode records in HES. The detailed ICD9, ICD10, and OPCS-4 we used to define T2D, CAD, IS, and HF are provided in Supplementary Table S1. CVD was defined as the union of these three subtypes. Analyses were restricted to subjects with T2D. Cases were identified as those who had onset of CVD at least 1 day later than T2D based on the HES records. The follow-up time was defined as the time duration between the T2D onset and the earliest CVD onset.

### PRS derivation

We calculated the PRS of CAD, IS, and HF using the corresponding largest-to-date GWAS summary statistics (Nikpay et al., 2015; Malik et al., 2018; Shah et al., 2020) and the linkage disequilibrium (LD) reference panel of 503 European samples from the 1000 Genomes Project phase III (Genomes Project Consortium et al., 2015) based on several state-of-the-art PRS methods [P+T (Chang et al., 2015), LDPred (Vilhjálmsson et al., 2015), PRS-CS (Ge et al., 2019), and AnnoPred (Hu et al., 2017)] as described previously (Ye et al., 2021).

To build the meta-PRS, we conducted a two-stage training. In the first stage, we used UKBB white British participants who did not have CVD records in the HES as the training dataset with the aim of selecting the best-performing PRS for the onset of each of the three CVD subtypes. In this training dataset, the cases were participants who self-reported CAD (IS/HF) history in an interview with a trained nurse but were without explicit HES CAD (IS/HF) onset record, and the controls were the remaining healthy population. In the second stage, we used a five-fold cross-validation strategy; where in each iteration, we randomly selected four-fifths of the participants with explicit HES T2D onset records as the training dataset. Based on the best-performing PRS of CAD, IS, and HF (named PRS_CAD_, PRS_IS_, PRS_HF_) selected in the first stage, the meta-PRS_CVD_ was built as follows:

$${meta-PRS}_{CVD,i}=\frac{\beta_{1}Z_{i1}+\beta_{2}Z_{i2}+\beta_{3}Z_{i3}}{\sqrt{\beta_{1}^{2}+\beta_{2}^{2}+\beta_{3}^{2}+2\beta_{1}\beta_{2}\rho_{1,2}+2\beta_{1}\beta_{3}\rho_{1,3}+2\beta_{2}\beta_{3}\rho_{2,3}}},$$

where $Z_{i1},Z_{i2},$ and $Z_{i3}$ were the standardized PRS_CAD_, PRS_IS_, and PRS_HF_ for the i th individual; $\beta_{1},\beta_{2},$ and $\beta_{3}$ were the regression coefficients of the Cox regression models on standardized PRS_CAD_, PRS_IS_, and PRS_HF_ for CVD occurrence among T2D patients; and $\rho_{m,n}$ was the Pearson correlation coefficient between $Z_{m}$ and $Z_{n}$ . Both regression coefficients and Pearson correlation coefficients were calculated as the mean values from five iterations in the training dataset as described before, the remaining one-fifth of the participants with explicit HES T2D onset records were then regarded as the testing dataset to evaluate the prediction performance of meta-PRS_CVD_ for CVD. The overall prediction performance was defined as the average of the results from the five iterations.

### Clinical risk factors

To evaluate the performance of meta-PRS_CVD_ when integrated with clinical variables, we collected a set of established clinical risk factors from two classic prediction models for CVD onset, including the Framingham Risk Model (Gordon et al., 1977) and the Pooled Cohort Risk Equations (PCE) (Muntner et al., 2014). Specifically, the Framingham Risk Model included age, sex, blood pressure, total cholesterol (TC), high-density lipoprotein cholesterol (HDL-C), and smoking (Gordon et al., 1977); the PCE included age, sex, ancestry, blood pressure, TC, HDL-C, and smoking (Muntner et al., 2014). Besides, evidence showed that hyperglycemia could be a potential explanation for CVD risk among T2D patients (Joseph et al., 2022).

Hence taking the overlaps between UKBB available data and risk factors as mentioned above, we included age of T2D onset, sex, body mass index (BMI), current smoking status, hypertension, TC, low-density lipoprotein cholesterol (LDL-C), HDL-C, Triglyceride (TG), cholesterol-lowering medication, and glucose level in our basket. Ancestry was not included since our analyses were all based on the white British European samples. The clinical risk model was then built using these clinical factors using the Cox regression model based on the training dataset as described in the second-stage training of meta-PRS_CVD._

### Statistical analysis

We applied the Cox regression models to assess the associations between four PRSs (meta-PRS_CVD_, PRS_CAD_, PRS_HF_, and PRS_IS_) and CVD occurrence among patients with T2D. Each model was adjusted for the age of T2D onset, sex, and the first 10 principal components. Follow-up started from the earliest onset date of T2D and was censored at CVD, death, or 5th February 2021, whichever was the earliest. During the first stage of the training of the meta-PRS_CVD_, we only calculated the area under the receiver operating characteristic curve (AUC) in the training set as the measurement to select the best-performing PRS for each subtype (PRS_CAD_, PRS_HF_, and PRS_IS_). Then in the testing set, both AUC and hazard ratios (HRs) with their 95% confidence intervals (CIs) were calculated to assess the prediction performance of different PRS and the integrated model combining meta-PRS_CVD_ with clinical risk factors.

We followed a strategy proposed in the Ripatti’s paper (Mars et al., 2020) to investigate the contributions of meta-PRS_CVD_ and clinical risks in developing CVD among T2D patients with different onset ages, where we first divided the patients into early- and late-onset groups with 55 years old of onset as the cut point. By defining the participants at the top 20% of meta-PRS as the high PRS risk group and those at the top 20% of predicted clinical risk score as the high clinical risk group, we then compared the proportions of individuals with high PRS or high clinical risk scores or both in each age group. Besides, we compared the absolute risk reduction (ARR) across the high PRS risk group and the remaining by calculating the differences in CVD incidence rate between early- and late-onset groups. All the statistical analyses were conducted in R (version 4.2.0). Statistical significance was set as 0.05 for each test.

## Results

### Population characteristics and clinical risk factors

Based on the disease defined in Supplementary Table S1, the demographics and baseline clinical information of the study subject are summarized in Table 1 and Supplementary Figure S1. A total of 21,092 T2D patients from UKBB were included in this study. Among these T2D patients, 4,015 developed CVD after 1 or more days following their T2D occurrence. Consistent with previous clinical research, the incidence rate of CVD was significantly higher in older patients; obesity, hypertension, hyperlipidemia, hyperglycemia, and ever smoking were all significantly associated with CVD occurrence among T2D patients.

**TABLE 1:** Baseline characteristics of T2D patients.

Characteristics	All T2D patients (*n* = 21,092)	T2D patients with CVD (*n* = 4,015)	HR (95% CI)	*p*-value
Age (years), *n* (%)^a^			1.04 (1.03–1.04)	<2e−16
≤55	3,589 (17%)	682 (17%)	—	—
>55	17,503 (83%)	3,333 (83%)	—	—
Sex, number of Male (%)	12,611 (60%)	2,845 (71%)	1.53 (1.41–1.66)	<2e−16
BMI, *n* (%)^a^			1.02 (1.02–1.03)	7.44e−14
<24	1,520 (7%)	186 (5%)	—	—
24–28	4,886 (23%)	796 (20%)	—	—
>28	14,686 (70%)	3,033 (76%)	—	—
Hypertension, *n* (%)	16,780 (80%)	3,525 (88%)	1.37 (1.24–1.53)	3.82e−09
TC mmol/L, mean (SD)	5.93 (1.28)	5.86 (1.36)	1.06 (0.93–1.21)	3.53e−01
TG mmol/L, mean (SD)	2.25 (1.27)	2.32 (1.3)	0.96 (0.92–1)	4.67e−02
HDL-C mmol/L, mean (SD)	3.93 (0.83)	3.95 (0.83)	1.02 (0.84–1.23)	1.75e−08
LDL-C mmol/L, mean (SD)	1.21 (0.33)	1.13 (0.3)	0.54 (0.44–0.67)	8.77e−01
Lipid lowering medication, *n* (%)	11,397 (54%)	2,814 (70%)	1.24 (1.12–1.35)	1.92e−06
Glucose mmol/L, mean (SD)	6.64 (2.95)	7.31 (3.45)	1.03 (1.02–1.04)	2.94e−11
Ever smoking, *n* (%)	11,919 (57%)	2,584 (64%)	1.27 (1.19–1.37)	4.47e−09

^a^ The hazard ratio (HR) per SD and *p*-value is calculated based on the continuous variables. CVD, cardiovascular disease; TC, total cholesterol; TG, triglyceride; HDL-C, high density lipoprotein cholesterol; LDL-C, low density lipoprotein cholesterol.

### Prediction performance of meta-PRS_CVD_

Among the four state-of-the-art PRS methods considered in our study (P+T, LDPred, PRS-CS, and AnnoPred), the empirical results based on the first-stage training dataset suggested that AnnoPred achieved the best onset prediction performance for all three CVD subtypes. These selected optimal PRSs were named PRS_CAD_, PRS_HF_, and PRS_IS_ (Supplementary Table S2), each of which involved around 3 million variants.

**FIGURE 1:**
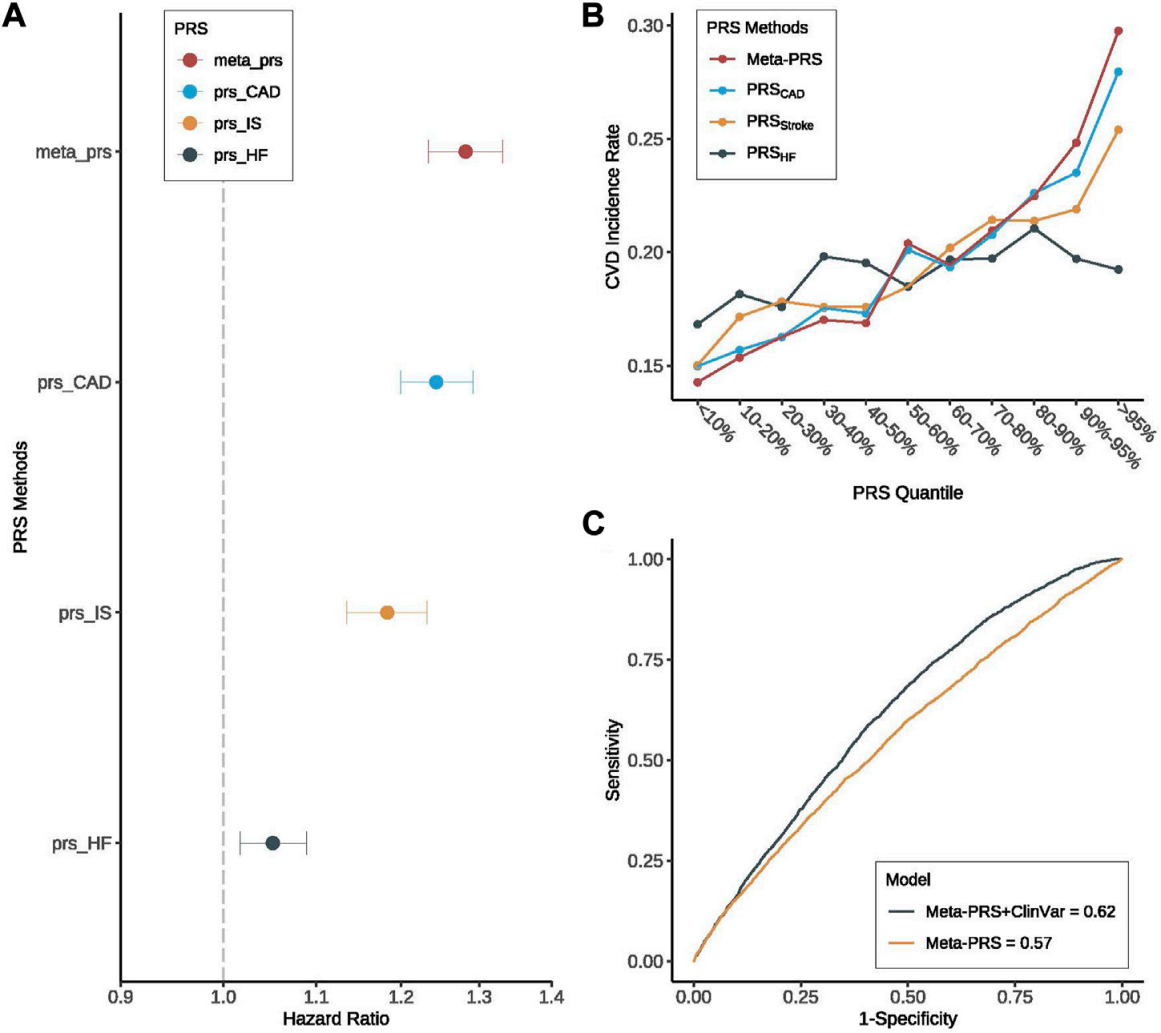
Prediction performance of meta-PRS_CVD_ for CVD among T2D patients. We compared the prediction performance of four PRSs (meta-PRS_CVD_, PRS_CAD_, PRS_Stroke,_ and PRS_HF_) for CVD among patients with T2D. **(A)** Hazard ratio (HR) increase per standard deviation was calculated for four PRSs through Cox regression models. The X-axis is HR increase per standard deviation, y-axis differentiate four PRSs, different colors indicate different PRSs. Among four PRSs, meta-PRS_CVD_ provided the highest HR for CVD. **(B)** Among T2D patients, we divided four PRSs into 10 quantiles and calculated the CVD incidence rate in each quantile. All four PRSs were able to stratify high-risk individuals, and meta-PRS_CVD_ was with the largest stratification capacity. **(C)** Based on meta-PRS_CVD_, two prediction models for CVD were compared with or without clinical variables, where the prediction accuracy was measured by ROC and AUC. And we found that while meta-PRS_CVD_ along can predict the risk of CVD with a high AUC, adding clinical variables could still improve the prediction performance by 8.8%.

For the prediction of CVD occurrence, meta-PRS_CVD_ was always better than the single PRS, with larger HRs and higher AUC (Figure 1; Table 2). Specifically, the HR per standard deviation (SD) of meta-PRS_CVD_ was 1.28 (95% CI: 1.23–1.33) and the AUC was 0.567 (95% CI: 0.544–0.589) when predicting CVD among T2D patients. T2D patients in the top 10% of meta-PRS_CVD_ had an incidence rate of 27.3%, which was 1.9 folds higher compared to T2D patients in the bottom 10% of meta-PRS_CVD_ (with an incidence rate of 14.3%). We further compared individuals with the top and bottom 10% of four PRSs through survival curves and the Cox proportional model. The survival curves for individuals with the top and bottom 10% meta-PRS_CVD_ were significantly different (Supplementary Figure S3). Consistently, the meta-PRS_CVD_ showed the highest adjusted HR of 1.43 (95% CI: 1.25–1.63, *p*-value = 2.73E-06), suggesting that patients at the top 10% had a 43% higher risk for CVD than patients in the bottom 10%. The model with clinical factors alone achieved an AUC of 0.612 (95% CI 0.59–0.635). And when integrating the meta-PRS_CVD_ with clinical factors, we built a full prediction model with even better prediction capacity with an AUC being 0.623 (95% CI 0.601–0.645). Additionally, for risk prediction of three CVD subtypes, the meta-PRS_CVD_ also showed the most significant AUC (Supplementary Table S3) and the highest HR (Supplementary Figure S2). Additionally, we established calibration models for four PRSs on CVD risk prediction and all four PRSs showed good calibration (Supplementary Figure S4).

**TABLE 2:** Prediction of CVD by different models of meta-PRS_CVD_ and separate PRS of each subtype.

Candidate PRSs\Models	PRS only^a^	PRS + baseline^b^
meta-PRS_CVD_	0.567 (0.544, 0.589)^c^	0.56 (0.538, 0.581)
PRS_CAD_	0.558 (0.536, 0.58)	0.555 (0.533, 0.576)
PRS_Stroke_	0.544 (0.522, 0.567)	0.545 (0.523, 0.566)
PRS_HF_	0.519 (0.497, 0.541)	0.533 (0.512, 0.554)

^a^ Model for CVD with corresponding PRS only.

^b^ Model for CVD with corresponding PRS, age of T2D onset, sex, and first 10 PCs.

^c^ The numbers in each entry are the area under curve (AUC): AUC (lower bound, upper bound); CAD, coronary artery disease; HF, heart failure.

### Contributions of meta-PRS_CVD_ and clinical risk in patients at different onset age

**FIGURE 2:**
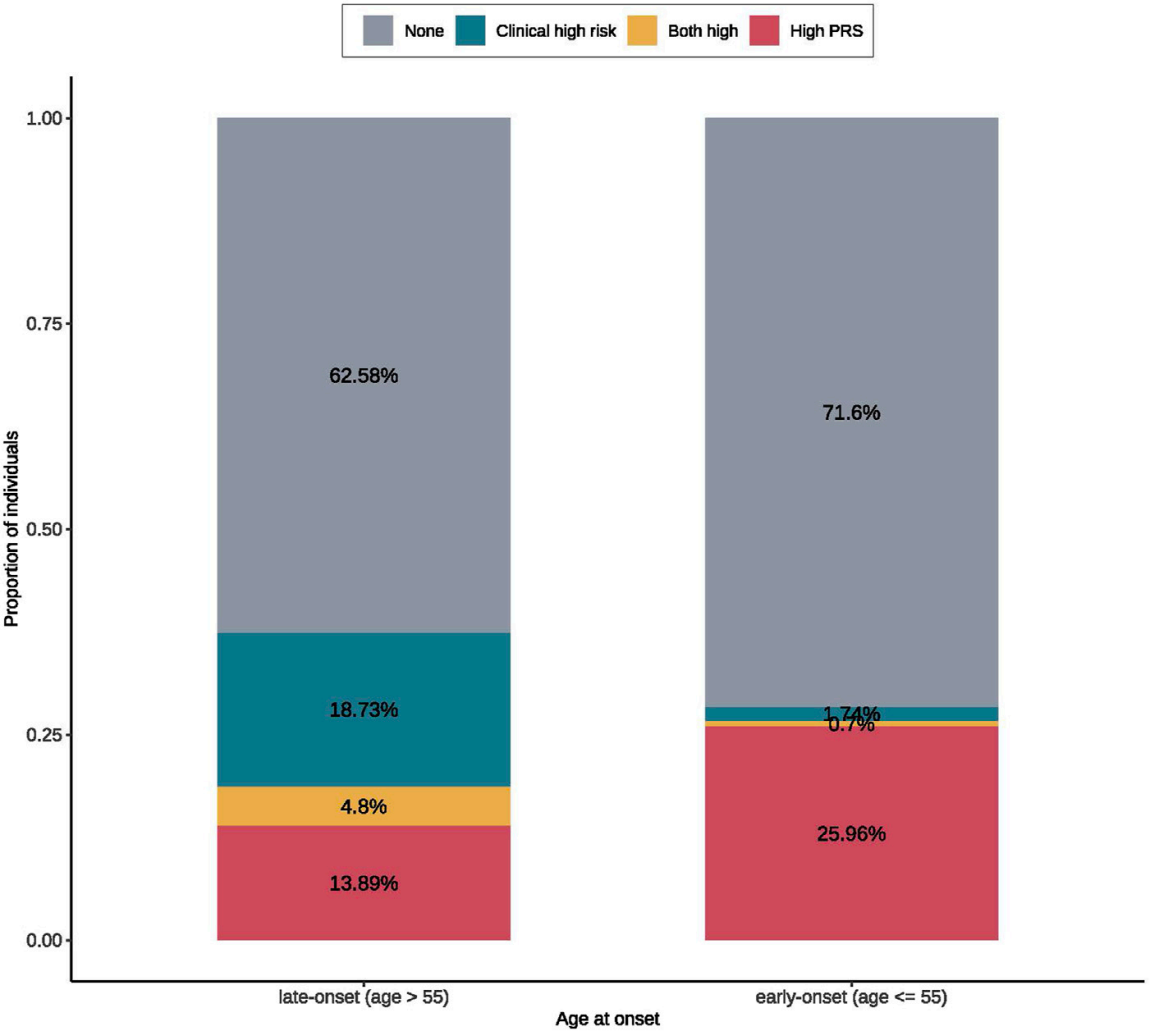
The proportions of early- and late-onset T2D cases with CVD in groups of high clinical/polygenic risk or neither. The high PRS was defined as the top 20% of the meta-PRS distribution. The clinical high risk was defined as the top 20% of the predicted risk score from the regression model for CVD with only clinical risk factors. According to the onset ages, patients were divided early- (≤55) and late-onset (>55) groups. In each onset age group, we calculated the proportions of patients with CVD that can be identified by PRS or clinical risk; where we found that early-onset group had a larger proportion of high PRS population than the late-onset group, while the late-onset group had a larger high clinical risk proportion than the early-onset group.

To compare the contributions of genetic risk and clinical risk to the risk of CVD across the onset ages, we summarized the proportions of T2D patients with the top decile meta-PRS_CVD_ or the top decile clinical risk scores or both in the early- (≤55) and late-onset (>55) groups. We observed that individuals with high meta-PRS_CVD_ accounted for 25.96% in the early-onset group, which was almost two folds higher than that in the late-onset group (13.89%). On the other hand, in the late-onset group, individuals with high clinical risk scores accounted for 18.73%, while in the early-onset group, the proportion was dramatically reduced to 1.74% (Figure 2). This indicates that the clinical risk factors had a stronger impact on the risk of CVD in the late-onset patients (>55), while in the early-onset patients, the risk of CVD was more driven by the genetic risk. Furthermore, we compared the distributions of PRS and clinical risk scores between CVD cases and controls among early- and late-onset groups (Supplementary Figure S5). The PRS could better distinguish cases and controls among early-onset groups compared to among late-onset groups (Supplementary Figures S3A, C).

**FIGURE 3:**
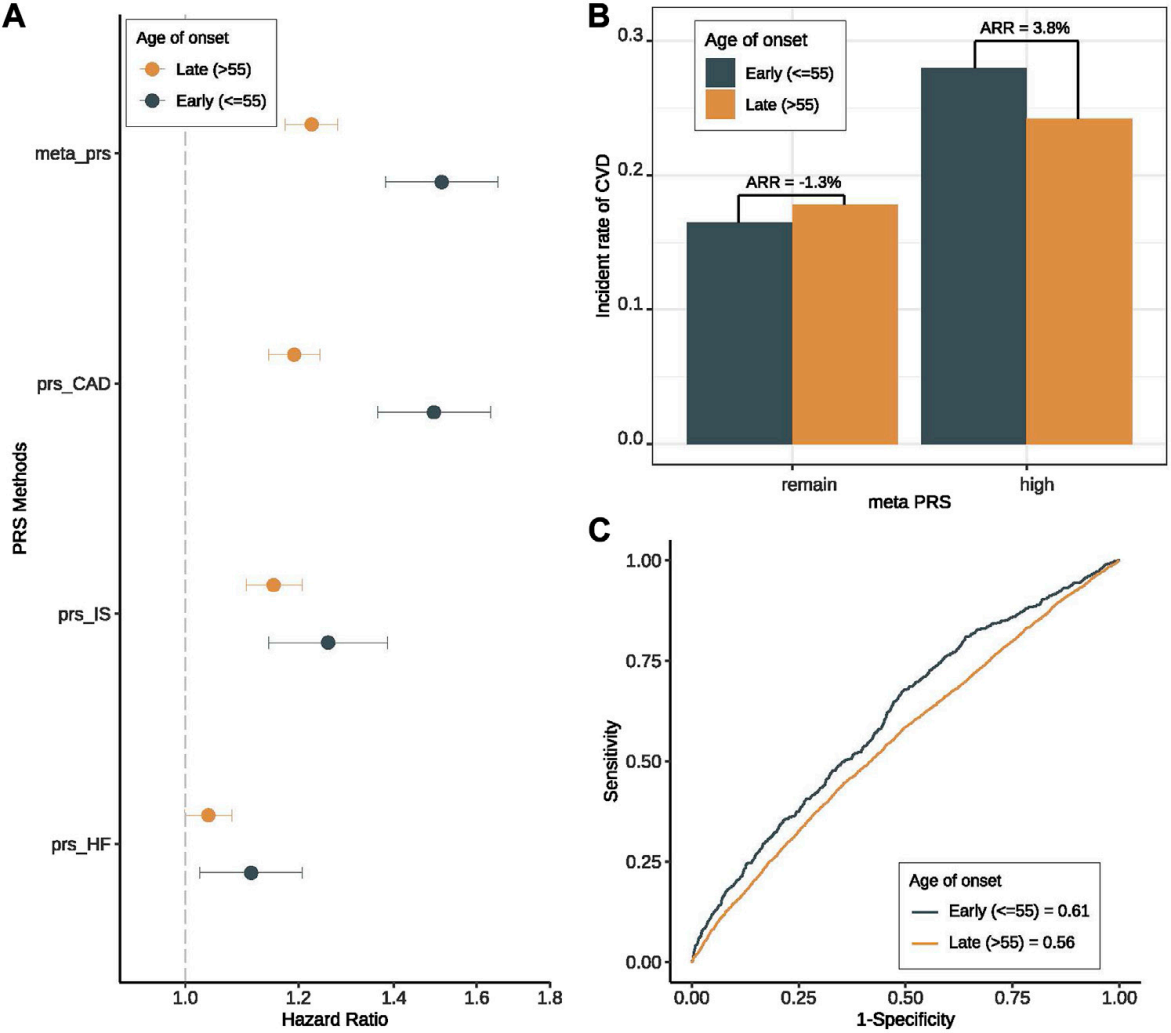
Prediction performance of meta-PRS for CVD among early- and late-onset T2D groups. We compared the prediction performance of four PRSs (meta-PRS_CVD_, PRS_CAD_, PRS_Stroke,_ and PRS_HF_) for CVD among patients with early- (onset age ≤ 55) and late-onset (onset age > 55) T2D. **(A)** Hazard ratio (HR) increase per standard deviation was calculated for four PRSs through Cox regression models. The X-axis is HR increase per standard deviation, y-axis differentiate four PRSs, different colors indicate different onset age groups. All four PRSs showed better prediction performance among early-onset group. **(B)** Based on the meta-PRS_CVD_ distribution, we divided all T2D patients into high (top 20%) and remaining subjects, and calculated the incidence rate for CVD in early- and late-onset groups respectively. The absolute risk reduction (ARR) was the difference in CVD incidence rate between two onset groups. We noticed that the ARR was higher in high-PRS group. **(C)** Based on meta-PRS, two prediction models for CVD among early- and late-onset T2D patients were compared, where the prediction accuracy was measured by ROC and AUC. Meta-PRS_CVD_ showed better prediction accuracy among early-onset T2D patients.

To further verify the important role of genetic risk in early-onset patients, we then investigated the prediction performance of PRSs for CVD in two groups of patients stratified by the onset age. And we observed that all the PRSs (including meta-PRS_CVD_ and single subtype PRSs) were more predictive in the young patients (onset age ≤ 55) compared to the old patients (onset age > 55) (Supplementary Table S4). It is worth noting that meta-PRS_CVD_ still performed the best in all settings. More specifically, when using meta-PRS_CVD_ to predict CVD risk among T2D patients, the HR for CVD based on young patients was 1.51 (95% CI 1.38–1.66) versus 1.23 (95% CI 1.18–1.28) among old patients (Figure 3A), and the AUC was 0.614 (95% CI 0.561–0.665) for early-onset patients and 0.557 (95% CI 0.532–0.581) for late-onset patients (Figure 3C). Adding the clinical variables further increase the AUC to 0.761 (96% CI 0.716–0.806) (Supplementary Figure S6). To further explore interactions between onset age and genetic risk, we compared the absolute risk reductions (ARRs) for CVD between high PRS and the remaining group. Within expectation, the ARR was 2.92 folds higher in the high-PRS group than in the remaining group (Figure 3B), showing that the meta-PRS_CVD_ can predict risk for CVD more accurately for early-onset CVD.

We also evaluated the prediction performances of four PRSs for CVD among male and female patients. No significant differences were identified (Supplementary Figure S7). One possible explanation was the insufficient power (Supplementary Figure S8).

## Discussion

In this study, we have developed a meta-PRS_CVD_ to predict the risk for major cardiovascular outcomes among T2D patients. By comprehensive analyses based on around 20,000 T2D patients, we showed that the newly-developed meta-PRS_CVD_ was able to effectively stratify the T2D patients into groups with different risks of CVD incidence. By combining the meta-PRS_CVD_ with established clinical variables, we further increased the prediction accuracy for CVD. Furthermore, our investigation of patients with different onset ages suggested that the risk of CVD among late-onset patients was more driven by clinical factors while the risk of CVD among early-onset patients was dominated by genetic risks.

Our results showed the superior prediction accuracy of AnnoPred over P+T, LDPred, and PRS-CS. This further validated the importance of biological annotation in generic risk prediction and was consistent with previous studies (Hu et al., 2017).

Previously, the use of PRS to predict cardiovascular events among T2D patients was not clear. Hong et al. (Hong et al., 2020) constructed PRS for CVD on 2,378 T2D patients and explored its prediction performance in the same dataset. One great limitation of this study was the lack of an independent validation dataset for PRS performance analysis, while in our study, we built the subtype PRSs using large-scale GWASs and tested the performance through cross-validation, which was more appropriate. Besides, the previous research (Hong et al., 2020) had a limited sample size (2,378) and number of SNPs (15, 47, and 231). We included around 20,000 T2D patients and over 3 million SNPs in our analysis, significantly boosting the prediction accuracy and statistical power.

We also showed that, with more advanced PRS algorithms, a larger study sample size, and by focusing on CVD events in the training design, the meta-PRS built in this study could achieve greater risk discrimination with the AUC being 0.56 (95% CI: 0.54–0.58).

Besides, our results also confirmed the significant role of clinical risk factors in the prediction of CVD events among T2D patients, which was shown in several previous studies (Hong et al., 2020). More specifically, the prediction accuracy of the meta-PRS_CVD_ was further increased to an AUC of 0.62 by combining with the clinical variables including hypertension, BMI index, high-density lipoprotein cholesterol, low-density lipoprotein cholesterol, total cholesterol content, smoking status, and whether to receive cholesterol treatment (Figure 1C).

In addition, we found that adding meta-PRS_CVD_ to the existing clinical risk factor model is more helpful for the clinical management of T2D patients with early-onset age in predicting the risk of CVD. In our population, 25.96% of early-onset (≤55) patients were at high risk of CVD predicted by meta-PRS_CVD_, much higher than 1.74% predicted by clinical variables, while clinical variables identified a larger proportion of high-risk subjects in the late-onset group (>55) (18.73% vs. 1.74%). Therefore, for T2D patients who are younger than 55, we may use PRS to predict their risk of CVD and to select the population which needs secondary prevention therapies accordingly, such as targeted lipid-lowering therapy and lifestyle modification for risk factor reduction, including a healthy diet, smoking cessation, exercise. And for patients who are 55 or older at the initial events, they would be better monitored using clinical risk variables for CVD risk predictions instead. Our results are consistent with a recent study (Mars et al., 2020), which suggested the impact of PRS on health outcomes would differ across different age groups.

We also note some limitations of the current work. First, since our meta-PRS_CVD_ was trained on the occurrence of three CVD subtypes (CAD, IS, and HF) among all subjects instead of among T2D patients, the difference between the endpoints of the original GWAS and our analysis may lead to potential information loss. The different definitions of diseases across cohorts may further exacerbate the information loss. Second, although we already have a larger sample size compared to previous studies, the sample size is still limited as the number of T2D cases who developed CVD events in the UKBB of the interested populations is rather small. Third, some cases included in the main analysis showed a short interval between T2D onset and CVD onset (less than 30 days), which might hinder the impact of T2D on CVD outcome and thus detriment the interpretations. We conducted further sensitivity analysis including 20,370 subjects with follow-up longer than 30 days. The prediction of PRSs for CVD was similar to our main analysis where the meta-PRS_CVD_ had the best performance (Supplementary Figure S9). The sensitivity analysis suggested that subjects had shorter follow-up time were not different or had little impact on our results. Finally, we did not incorporate variables such as ancestry, psychosocial factors, comorbidities of CVD including chronic kidney disease (CKD), peripheral artery disease (PAD), and chronic obstructive pulmonary disease (COPD) in our prediction models. Specifically, we used the white British European population from UKBB to establish the meta-PRS_CVD_ model, while the classical prediction models we referred to for clinical risk factors (Framingham Risk Model, Pooled Cohort Risk Equations) were based upon the US population. Further analyses are necessary to dissect the potential roles of these factors in CVD occurrence among T2D patients.

## Conclusion

In summary, we constructed a meta-PRS_CVD_ that can effectively predict the risk of severe cardiovascular diseases among T2D patients. Our results highlight the great potential of meta-PRS_CVD_ in secondary prevention among early-onset T2D patients.

## Data Availability

The data analyzed in this study is subject to the following licenses/restrictions: Because of the sensitive nature of the individual-level data collected for this study, requests to access the dataset from qualified researchers trained in human subject confidentiality protocols may be sent to UK Biobank (UKBB) at https://www.ukbiobank.ac.uk/register-apply. The summary-level data (e.g., PRS weights) are available from the corresponding author (hongyu.zhao@yale.edu) upon request. Requests to access these datasets should be directed to https://www.ukbiobank.ac.uk/register-apply.

## Abbreviations

PRS, polygenic risk score; CVD, cardiovascular disease; T2D, type 2 diabetes; CAD, coronary artery disease; AUC, area under the receiver operating characteristic curve; IS, ischemic stroke; HF, heart failure; GWAS, genome-wide association study; SNP, single nucleotide polymorphism; UKBB, UK Biobank; UK, United Kingdom; HES, hospital episode statistics; ICD9/10, 9th/10th version of international classification of diseases; OPCS-4, Population, Census and Census Office: Classification of Interventions and Procedures, Version 4; PC, principal component; LD, linkage disequilibrium; PCE, Pooled Cohort Risk Equations; TC, total cholesterol; HDL-C, high-density lipoprotein cholesterol; BMI, body mass index; LDL-C, low-density lipoprotein cholesterol; TG, triglyceride; HR, hazard ratio; CI, confidence interval; ARR, absolute risk reduction; SD, standard deviation; CKD, chronic kidney disease; PAD, peripheral artery disease; COPD, chronic obstructive pulmonary disease.
