# Supplementary material for: Genomic risk prediction of cardiovascular diseases among type 2 diabetes patients in the UK Biobank
Source: Front Bioinform. 2024 Jan 4;3:1320748. doi: 10.3389/fbinf.2023.1320748 (PMC10794561; doi:10.3389/fbinf.2023.1320748)
Supplement: Supplementary file 1 [file Table1.docx]

**Supplemental Tables**

## Supplemental Table 1. Diagnosis of diseases in the UK Biobank.

**Supplemental Table 2.** Prediction of state-of-art PRS methods among training samples.

**Supplemental Table 3.** Prediction of PRS for CVD subtypes

## Supplemental Table 4. Prediction of PRS for CVD in early and late onset T2D patients.

**Supplemental Table 1.** Diagnosis of diseases in the UK Biobank.

| **Disease** | **ICD-9** | **ICD-10** | **OPCS-4** |
| --- | --- | --- | --- |
| Coronary Artery Disease | 410-412 | I21-I23, I241, I252 | K40.1-40.4, K41.1-41.4, K45.1-45.5, K49.1-49.2, K49.8-49.9, K50.2, K75.1-75.4, K75.8-75.9 |
| Heart Failure | 428, 425.4 | I110, I130, I132, I255, I420, I425, I428-429, I50 | - |
| Ischemic Stroke | 430, 431, 434, 436 | I60-I64 | - |
| Type 2 Diabetes | - | E11 | - |

## Supplemental Table 2. Prediction of state-of-art PRS methods among training samples.

| CVD subtypes\PRS Methods | P+T | LDPred | PRS-CS | AnnoPred |
| --- | --- | --- | --- | --- |
| CAD | 0.617 (0.609, 0.625) | 0.628 (0.620, 0.636) | 0.632 (0.624, 0.641) | 0.643 (0.635, 0.651) |
| Stroke | 0.517 (0.494, 0.539) | 0.51 (0.487, 0.532) | 0.513 (0.49, 0.535) | 0.517 (0.495, 0.539) |
| HF | 0.512 (0.49, 0.533) | 0.537 (0.515, 0.559) | 0.512 (0.49, 0.533) | 0.538 (0.515, 0.56) |

The numbers in each entry are the area under curve (AUC): AUC (lower bound, upper bound); PRS: polygenic risk score; CVD: cardiovascular disease; CAD: coronary artery disease; HF: heart failure.

## Supplemental Table 3. Prediction of PRS for CVD subtypes.

| PRSs\CVD subtypes | CAD (2623/18469) | Stroke (909/20183) | HF (1989/19103) |
| --- | --- | --- | --- |
| Meta-PRS_CVD_ | 0.598 (0.573,0.625) | 0.523 (0.48,0.566) | 0.558 (0.528,0.588) |
| PRS_CAD_ | 0.597 (0.571,0.623) | 0.508 (0.466,0.55) | 0.545 (0.515,0.575) |
| PRS_Stroke_ | 0.548 (0.522,0.575) | 0.542 (0.499,0.585) | 0.55 (0.52,0.58) |
| PRS_HF_ | 0.518 (0.492,0.544) | 0.515 (0.473,0.556) | 0.524 (0.495,0.554) |

The numbers in the column’s headers are the sample size (number of patients with / without recurrence). The numbers in each entry are the area under curve (AUC): AUC (lower bound, upper bound); PRS: polygenic risk score; CVD: cardiovascular disease; CAD: coronary artery disease; HF: heart failure.

## Supplemental Table 4. Prediction of PRS for CVD in early and late onset T2D patients.

| PRSs\onset age | Late (3333/14170) | Early (682/2907) |
| --- | --- | --- |
| Meta-PRS_CVD_ | 0.557 (0.532,0.581) | 0.614 (0.561,0.665) |
| PRS_CAD_ | 0.549 (0.525,0.573) | 0.604 (0.551,0.657) |
| PRS_Stroke_ | 0.54 (0.516,0.565) | 0.566 (0.514,0.618) |
| PRS_HF_ | 0.516 (0.492,0.54) | 0.534 (0.481,0.586) |

The numbers in the column’s headers are the sample size (number of patients with / without recurrence). The numbers in each entry are the area under curve (AUC): AUC (lower bound, upper bound); PRS: polygenic risk score; CVD: cardiovascular disease; CAD: coronary artery disease; HF: heart failure.

# Supplemental Figure Legends

## Figure S1. Distribution of age at onset and follow-up time

We checked the distribution of age at T2D onset (A) and follow-up time for CVD (B) in T2D patients. Cases had similar age range to controls and had shorter and less centralized follow-up time.

**Figure S2. Hazard ratios of four PRSs for CVD subtypes**

The associations between four PRSs and three CVD subtypes were examined through Cox proportional hazards models. We noticed that meta-PRS showed the highest HRs for CAD and HF, and the second best for IS.

**Figure S3. Survival curves for CVD stratified by four PRSs**

We dichotomized four PRSs and assessed their associations with survival probability for CVD. The difference in survival experiences was most significant when dichotomized by meta-PRSCVD (A).

**Figure S4. Calibration of four PRSs for CVD**

We established calibration models with four PRSs on CVD. All four PRSs showed great calibration for CVD risk predictions.

**Figure S5. Distribution of PRS and clinical risk score among early- and late-onset groups**

We compared the distributions of meta-PRS (A)/clinical risk score (B) across four groups defined by end age of follow-up and CVD status. Both scores can distinguish CVD cases from controls. The early-onset CVD showed the highest mean for meta-PRS while the late-onset CVD had the highest mean for clinical risk score.

**Figure S6. Prediction performance of PRS and clinical factors among early-onset group**

We explored the prediction performance (AUC) of two models (meta-PRS_CVD_ alone, and combination of meta-PRS_CVD_ and clinical variables) among early-onset group. Compared with prediction among all subjects (Figure 1-C), the AUCs among early-onset group were 1.23-folds for combined model and 1.07-folds for meta-PRS_CVD._

**Figure S7.** **Contributions of PRSs for CVD among male and female T2D patients.**

We explored the prediction performance of four PRSs (meta-PRS_CVD_, PRS_CAD_, PRS_IS_ and PRS_HF_) for CVD among male and female T2D patients respectively. No significant differences were found in HR (A) or AUC (B).

**Figure S8. Simulation of power to detect interaction between PRS and sex**

We conducted simulation analysis with different sample size to explore the power the analysis for interaction between PRS and sex. Even the largest sample size in the simulation (N=50,000) showed an insufficient power (12%), which explained the reason we failed to identify the interaction.

**Figure S9. Sensitivity analysis**

We conducted sensitivity analyses among subject with follow-up time longer than 30 days. The results were similar to the main analysis, suggesting subjects with shorter follow-up time were the same as other subjects.

## Supplemental Figure 1. Distribution of age at T2D onset and CVD follow-up time

We checked the distribution of age at T2D onset (A) and follow-up time for CVD (B) in T2D patients. Cases had similar age range to controls and had shorter and less centralized follow-up time.

**Supplemental Figure 2.** Hazard ratios of four PRSs for CVD subtypes

The associations between four PRSs and three CVD subtypes were examined through Cox proportional hazards models. We noticed that meta-PRS showed the highest HRs for CAD and HF, and the second best for IS.

**Supplementary Figure 3. Survival curves for CVD stratified by PRSs**

We compared the survival experiences between individuals with top 10% and bottom 10% genetic risk across four PRSs. The difference in survival experiences was most significant when stratified by meta-PRS_CVD_ (A).

**Supplementary Figure 4. Calibration of four PRSs for CVD**

We established calibration models with four PRSs on CVD. All four PRSs showed great calibration for CVD risk predictions.

**Supplementary Figure 5.** Distribution of PRS and clinical risk score among early- and late-onset groups

We compared the distributions of meta-PRS (A and C)/clinical risk score (B and D) between cases and controls among early- and late-onset groups. The means (standard deviations) of meta-PRS were 0.379 (1.036) for CVD cases and -0.038 (1.011) for controls in early-onset group, and 0.153 (1.009) for CVD cases and -0.046 (0.986) for controls in late-onset group. The means (standard deviations) of clinical risk score were -0.258 (0.745) for CVD cases and -1.096 (1.021) for controls in early-onset group, and 0.452 (0.766) for CVD cases and 0.127 (0.895) for controls in late-onset group. Both scores can distinguish CVD cases from controls.

**Supplementary Figure 6.** Prediction performance of PRS and clinical factors among early-onset group

We explored the prediction performance (AUC) of two models (meta-PRS_CVD_ alone, and combination of meta-PRS_CVD_ and clinical variables) among early-onset group. Compared with prediction among all subjects (Figure 1-C), the AUCs among early-onset group were 1.23-folds for combined model and 1.07-folds for meta-PRS_CVD._

**Supplementary Figure 7.** Contributions of PRSs for CVD among male and female T2D patients.

We explored the prediction performance of four PRSs (meta-PRS_CVD_, PRS_CAD_, PRS_IS_ and PRS_HF_) for CVD among male and female T2D patients respectively. No significant differences were found in HR (A) or AUC (B).

**Supplementary Figure 8.** Simulation of power to detect interaction between PRS and sex

We conducted simulation analysis with different sample size to explore the power the analysis for interaction between PRS and sex. Even the largest sample size in the simulation (N=50,000) showed an insufficient power (12%), which explained the reason we failed to identify the interaction.

**Supplementary Figure 9.** Sensitivity analysis.

We conducted sensitivity analyses among subject with follow-up time longer than 30 days. The results were similar to the main analysis, suggesting subjects with shorter follow-up time were the same as other subjects. (A) Hazard ratio (HR) increase per standard deviation was calculated for four PRSs through Cox regression models. Among four PRSs, meta-PRS_CVD_ provided the highest HR for CVD. (B) Among T2D patients, we divided four PRSs into ten quantiles and calculated the CVD incidence rate in each quantile. All four PRSs were able to stratify high-risk individuals, and meta-PRS_CVD_ was with the largest stratification capacity. (C) Based on meta-PRS_CVD_, two prediction models for CVD were compared with or without clinical variables, where the prediction accuracy was measured by ROC and AUC. And we found that while meta-PRS_CVD_ along can predict the risk of CVD with a high AUC, adding clinical variables could still improve the prediction performance by 8.8%, which was the same as our primary findings in Figure 1.
